# Valproic acid inhibits tumor angiogenesis in mice transplanted with Kasumi-1 leukemia cells

**DOI:** 10.3892/mmr.2013.1834

**Published:** 2013-11-28

**Authors:** ZHI-HUA ZHANG, CHANG-LAI HAO, PENG LIU, XIA TIAN, LI-HONG WANG, LEI ZHAO, CUI-MIN ZHU

**Affiliations:** 1 Affiliated Hospital of Chengde Medical College, Chengde, Hebei 067000, P.R. China; 2 The First Hospital of Shijiazhuang City, Shijiazhuang, Hebei 050000, P.R. China; 3 Chinese PLA 89 Hospital, Weifang, Shandong 261000, P.R. China

**Keywords:** histone deacetylase, valproic acid, Kasumi-1 cells, tumor angiogenesis

## Abstract

Histone deacetylase (HDAC) inhibitors have been reported to inhibit tumor angiogenesis via the downregulation of angiogenic factors. Our previous *in vitro* studies demonstrated that valproic acid (VPA) exerted antitumor effects on Kasumi-1 cells, which are human acute myeloid leukemia cells with an 8;21 chromosome translocation. In the present study, the effects of VPA on tumor angiogenesis were investigated in mice transplanted with Kasumi-1 cells. Semi-quantitative reverse transcription-polymerase chain reaction, western blotting and immunohistochemistry were used to detect the expression of vascular endothelial growth factor (VEGF), VEGF receptor (VEGFR2) and basic fibroblast growth factor (bFGF). The tumor microvessel density was measured following staining with an anti-CD34 antibody. Chromatin immunoprecipitation was used to study the effect of VPA-induced histone hyperacetylation on *VEGF* transcription. An intraperitoneal injection of VPA inhibited tumor growth and angiogenesis in mice transplanted with Kasumi-1 cells. The mRNA and protein expression of VEGF, VEGFR2 and bFGF were inhibited by VPA treatment. In addition, VPA downregulated HDAC, increased histone H3 acetylation and enhanced the accumulation of hyperacetylated histone H3 on the VEGF promoters. The findings of the present study indicate that VPA, an HDAC inhibitor, exerts an antileukemic effect through an anti-angiogenesis mechanism. In conclusion, the mechanism underlying VPA-induced anti-angiogenesis is associated with the suppression of angiogenic factors and their receptors. VPA may increase the accumulation of acetylated histones on the VEGF promoters, which possibly contributes to the regulation of angiogenic factors.

## Introduction

Chromosomal translocations are frequent events that occur in leukemia. The translocation t(8;21)(q22;q22) is one of the most frequent chromosomal translocations in leukemia and accounts for 12–15% of acute myeloid leukemia (AML) and ~40–50% of M2 AML (French-American-British classification) (1). This translocation involves the *AML1* gene at q22 on chromosome 21 and the *ETO* gene at q22 on chromosome 8, resulting in an *AML1/ETO* fusion gene. This fusion gene encodes a chimeric protein, AML1/ETO. The chimeric protein silences target gene transcription by recruiting histone deacetylases (HDACs), which remove acetyl groups from histone lysine residues. The abnormal recruitment of HDAC due to chromosomal rearrangements often occurs in the development of malignant tumors and contributes to their pathogenesis (2). Several studies have demonstrated that the abnormal AML1/ETO protein and the silencing of hematopoietic genes contribute to the hematopoietic developmental abnormalities of AML with t(8;21) (3–6). Inhibition of HDAC activity has been reported to restore the abnormal histone acetylation in tumors, thus resulting in the growth arrest, differentiation and/or apoptotic cell death of tumor cells (7). Therefore, HDAC inhibitors represent a promising treatment for patients with AML with t(8;21), as they may enhance histone acetylation via inhibition of HDAC activities, thus restoring the disrupted gene transcripts in AML (8).

Angiogenesis is critical for tumor growth and metastasis. Vascular endothelial growth factor (VEGF), VEGF receptors (VEGFRs) and basic fibroblast growth factors (bFGFs) are the most potent pro-angiogenic factors and are critical in tumor angiogenesis (9,10). Anti-angiogenic approaches are a novel strategy to treat AML. It has been reported that the HDAC inhibitor, FK228, inhibits the expression of angiogenic factors, including VEGF and bFGF, in PC-3 xenografts implanted in nude mice, indicating that the antitumor effects of FK228 are mediated through the inhibition of angiogenesis (11). Valproic acid (VPA), which is widely used clinically for the treatment of epilepsy, has been demonstrated to be a strong HDAC inhibitor (12). Our previous *in vitro* studies revealed that VPA exerted antitumor effects on Kasumi-1 cells, human acute myeloid leukemia cells with an 8;21 chromosome translocation, via downregulation of VEGF and VEGFR (13,14). The purpose of the present study was to investigate the effect of VPA on tumor growth and the expression of angiogenic factors in mice transplanted with Kasumi-1 cells, and also to analyze the histone acetylation on VEGF promoters in these cells.

## Materials and methods

### Tumor cells and animals

The Kasumi-1 cell line was a gift from Dr Jianxiang Wang at the Institute of Hematology, Chinese Academy of Medical Science (Tianjin, China). The cells were maintained in culture with RPMI-1640 medium supplemented with 20% fetal bovine serum in a 37°C incubator with 5% CO_2_ and 95% humidity. Female BALB/c nude mice (SPF grade; 10–15 g; 4–6 weeks old) were purchased from Beijing Vital River Lab Animal Technology Co., Ltd. (Beijing, China). The study was approved by the Chengde Medical College Animal Research Ethics Committee.

### Tumor generation and VPA treatment

Splenectomies were performed on the BALB/c nude mice. One week after the splenectomies, the mice received whole body irradiation with ^137^Cs at a dose of 4 Gy. At 48–72 h post-irradiation, the mice were subcutaneously implanted with Kasumi-1 cells (2×10^7^ cells/mouse with 0.15–0.2 ml) in the right axillary region. The mice were randomly assigned to two groups, the VPA (n=6) and control (n=6) groups. When the tumors were ~200 mm^3^ in size at ~10 days post-implantation, 0.2 ml VPA (500 mg/kg body weight) or 0.2 ml saline was injected intraperitoneally every day. VPA (Sigma-Aldrich, St. Louis, MO, USA) was dissolved in saline at a concentration of 25 mg/ml. The longest diameter (a) and the shortest diameter (b) of the tumor were measured every three days, and the tumor volume (TV) was calculated according to the following formula: TV = 1/2 × a × b^2^. The relative TV (RTV) was calculated as the ratio between the TV on day N and the TV on the day of injection (day 0) according to the following formula: RTV = (TV on day N) / (TV on day 0). The tumor growth inhibition rate (IR) was calculated from the following formula: IR (%) = [1 − RTV_(VPA group)_ / RTV_(control)_) × 100, where RTV_(VPA group)_ is the RTV in the VPA-treated group and RTV_(control)_ is the RTV in the control group. Following two weeks of injections, the mice were sacrificed by cervical dislocation and the tumor masses were removed for the following experiments.

### Immunohistochemistry

The tumor masses were fixed with 10% formalin and embedded with paraffin. Tissue sections (5-μm thick) were obtained from paraffin-embedded tissue blocks. The tissue sections were immunohistochemically stained for CD34, VEGF, VEGFR2 and bFGF. Briefly, the sections were washed in xylene to remove the paraffin, rehydrated with serial dilutions of alcohol and then washed in phosphate-buffered saline. The samples were then incubated in primary antibodies against CD34, VEGF, VEGFR2 and bFGF overnight at 4°C. All primary antibodies were rat anti-human antibodies (Santa Cruz Biotechnology, Inc., Santa Cruz, CA, USA). Subsequent to the primary antibody being washed off, the biotinylated goat anti-rat IgG secondary antibody (Santa Cruz Biotechnology, Inc.) was applied and then reacted with horseradish peroxidase-conjugated streptavidin. The sections were stained with diaminobenzidine solution and counterstained with hematoxylin.

### Tumor microvessel density (MVD) analysis performed following staining with the anti-CD34 antibody

Microvessels with brownish staining in the cytoplasm of the endothelium were included. A single endothelial cell was counted as a single vessel. An endothelial cell cluster with a branching structure, which was clearly separated from the adjacent endothelial cells, was also counted as a single vessel. At a low-power field (x40), the tissue sections with the most intense vascular density were selected. At a high-power field (x200), microvessels in five fields were counted in the areas with the most intense vascular density. The mean microvessel count of the five most vascular areas was used as the MVD.

### Semi-quantitative reverse transcription-polymerase chain reaction (RT-PCR)

Total RNA was isolated from the tumor masses using TRIzol reagent (Invitrogen, Life Technologies, Carlsbad, CA, USA) according to the manufacturer’s instructions. The final RNA concentration was adjusted to 1 μg/μl. RNA was reverse transcribed into complementary DNA using the reverse transcriptase of Moloney murine leukemia virus (Bio Basic Canada Inc., Markham, ON, Canada). PCR was performed using Taq DNA polymerase [Takara Biotechnology, Co., Ltd., Dalian, China]. The primers were as follows: Sense: 5′-GAAGTGGTGAAGTTCATGGATGTC-3′ and antisense: 5′-CGATCGTTCTGTATCAGTCTTTCC-3′ for VEGF (size, 260 bp); sense: 5′-AGAGCGACCCTCACATCAAG-3′ and antisense: 5′-TCGTTTCAGTGCCACATACC-3′ for bFGF (size, 224 bp); and sense: 5′-GGGGATTGACTTCAACTGG-3′ and antisense: 5′-GACCCTGACAAATGTGCTG-3′ for VEGFR2 (size, 211 bp). β-actin (size, 453 bp) was used as an internal control. The reaction conditions consisted of 38 cycles of 95°C for 45 sec, 61°C for 45 sec and 72°C for 60 sec. The PCR products were analyzed by electrophoresis on a 1.8% agarose gel containing ethidium bromide, and the gel was visualized with a digital imaging system (Fujifilm; Fuji, Tokyo, Japan). The relative mRNA expression of VEGF, bFGF and VEGFR2 was normalized to the β-actin concentration.

### Western blotting

For western blotting, the tumor masses were homogenized on ice in RIPA lysis buffer [50 mM Tris-HCl, 150 mM NaCl and 1% NP-40 (pH 7.4)]. Nuclear proteins were extracted using the Nucleoprotein Extraction kit (Sangon Biotech, Co., Ltd., Shanghai, China) according to the manufacturer’s instructions. The protein concentrations were determined with the bicinchoninic acid method. Equal quantities of proteins were loaded and separated by electrophoresis in 120 g/l SDS-PAGE and then transferred onto polyvinylidene fluoride membranes. The membranes were incubated with primary antibodies against HDAC1 (rabbit anti-human polyclonal antibodies; Proteintech Group, Chicago, IL, USA) or acetylated histone H3 (Ac-H3; rabbit anti-human monoclonal antibodies; Epitomics, Burlingham, CA, USA) at 4°C overnight. Blots were stained with horseradish peroxidase-linked goat anti-rabbit Ig secondary antibodies and developed with a chemiluminescence detection system (Fujifilm). The nuclear protein, lamin B served as a loading control.

### Chromatin immunoprecipitation (ChIP)

ChIP was performed using the ChIP assay kit (Upstate Biotechnology, Billerica, MA, USA) according to the manufacturer’s instructions. Briefly, tumor masses were cut into small sections (Xinzhi Co. Ltd., Ningbo, China) and treated with 1% formalin for 10 min at room temperature. The tissues were then homogenized with lysis buffer and sonicated five times at 80 W for 10 sec/time, at 60 sec intervals. Following centrifugation (5,000 × g, 1 min), the supernatant was removed, and 20 μl supernatant was saved for the controls (DNA input). Rabbit anti-human monoclonal antibodies against Ac-H3 (3 μg) were added into the supernatant and incubated overnight at 4°C with gentle rotation. Antibodies against RNA polymerase were used as a positive control and mouse IgG was used as a negative control. Protein G agarose beads were added into the solution, and the samples were incubated at 4°C for 1 h. The beads were washed five times with buffers according to the the ChIP assay kit protocol. The protein-DNA complex was eluted with an elution buffer containing 20% SDS and 1M NaHCO_3_. The immunoprecipitated chromatin was dissolved in 0.9% saline and then incubated at 65°C for 4 h to reverse the cross-linking and separate the protein-DNA complex. The DNA was extracted, and RT-PCR was performed with primers specific for the VEGF promoter. The primers were as follows: Sense: 5′-CTT CGA GAG TGA GGA CGT GTG T-3′ and antisense: 5′-GGA GCA GGA AAG TGA GGT TAC G-3′ for P1; sense: 5′-CCA GAC TCC ACA GTG CAT ACG T-3′ and antisense: 5′-TGGGAC TGG AGT TGC TTC ATG-3 for P2; sense: 5′-TGC TGC ATT CCC ATT CTC AGT-3′ and antisense: 5′-ATC TTC CCTAAG TGC TCC CAA AG-3′ for P3; sense: 5′-CAG GGA AAG GAT GAT CAC TGT CA-3′ and antisense: 5′-TGC CTT TCA CCA GGA CAA AGT-3′ for I1; and sense: 5′-ATG GAT GTC TAT CAG CGC AGCT-3′ and antisense: 5′-TGG TGA TGT TGG ACT CCTCAG T-3′ for E3. The reaction conditions were 94°C for 3 min, followed by 32 cycles of 94°C for 20 sec, 57°C for 30 sec and 72°C for 30 sec and a final extension of 72°C for 2 min.

### Statistical analysis

Statistical analyses were performed using SPSS 17.0 (SPSS, Inc., Chicago, IL, USA). All the data presented are based on an average of at least three independent experiments. The values are presented as the mean ± standard deviation. Student’s t-test’s were used to compare the differences between groups. P<0.05 was used to indicate a statistically significant difference.

## Results

### VPA inhibits tumor growth in mice transplanted with Kasumi-1 cells

**Figure 1:**
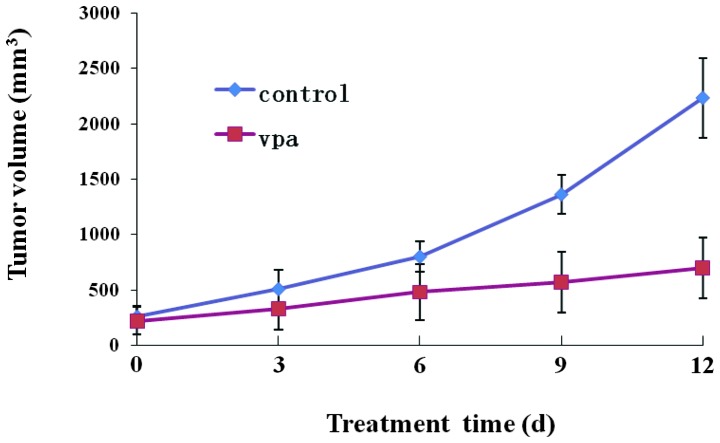
Time-course of the tumor growth in mice transplanted with Kasumi-1 cells. The mice were intraperitoneally administered 0.2 ml VPA (500 mg/kg body weight) or 0.2 ml saline daily for two weeks. The tumor volumes (TV) were measured every three days. The data are shown as the mean ± standard deviation (n=6 for the control and VPA groups). VPA, valproic acid.

**Figure 2:**
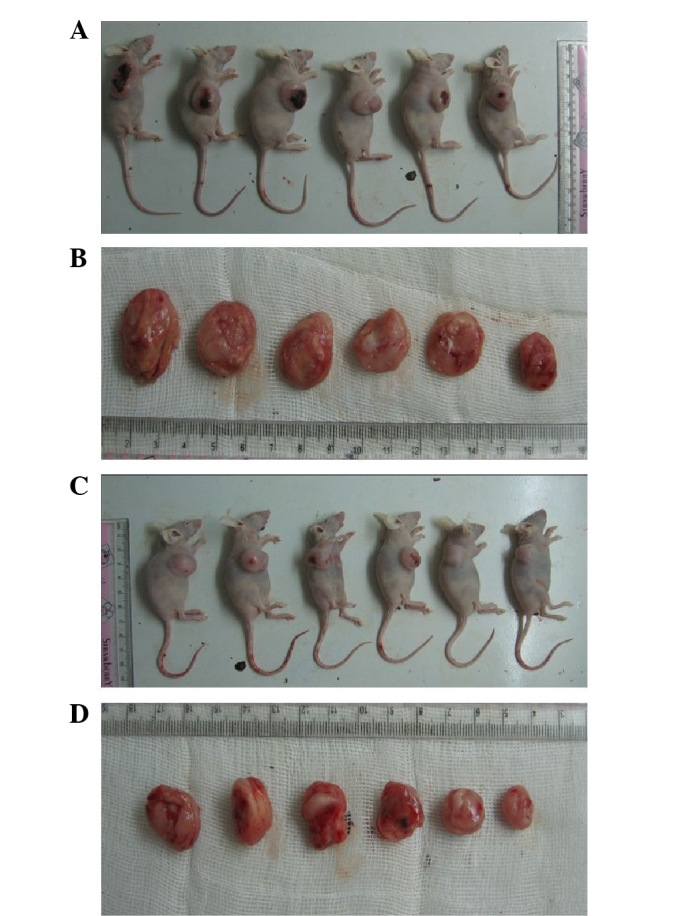
Effects of VPA on tumor morphology following sacrifice of the mice. The figures show the tumors in (A) the control mice and (B) after removal, and tumors in (C) VPA-treated mice and (D) after removal. VPA, valproic acid.

The effect of VPA on tumor growth was investigated in mice transplanted with Kasumi-1 cells. None of the mice died prior to sacrifice at the end of the experiments. The size of the tumor was measured every three days following daily injection of VPA for two weeks. Fig. 1 shows the time-course of tumor growth in the control and VPA-treated groups. The TV in the VPA group increased with time more slowly than that in the control group. The final TV in the VPA group was 699.4±271.01 mm^3^, which was significantly less than the TV of 2235.0±360.21 mm^3^ observed in the control group (P<0.05; Table I). Following sacrificing the mice, the size, weight and RVT of the tumors in the VPA group were significantly less than those in the control group (P<0.05; Table I; Figs. 1 and 2), indicating that VPA inhibited the tumor growth in the mice transplanted with Kasumi-1 cells. The IR rate in the VPA group was 57.25% at the end of the experiment.

### VPA inhibits tumor angiogenesis in mice transplanted with Kasumi-1 cells

**Figure 3:**
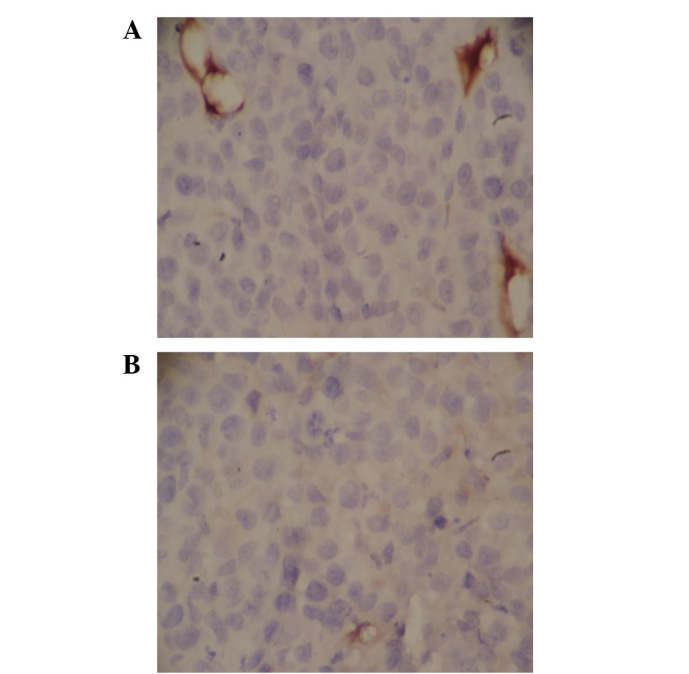
Staining of microvessels by anti-CD34 immunostaining (brownish staining) in (A) the control group and (B) the VPA-treated group. VPA, valproic acid.

The effect of VPA on tumor angiogenesis was tested in mice transplanted with Kasumi-1 cells by measuring MVD using CD34 immunostaining, which has been used in previous studies (15,16). Specific staining of capillary-like vessels by anti-CD34 was observed in the control and VPA groups (Fig. 3). The mean MVD in the VPA group (12.23±4.11; number of microvessels) was significantly lower than that in the control group (32.59±5.76) (P<0.05), indicating that VPA inhibited angiogenesis in the mice transplanted with Kasumi-1 cells.

### VPA inhibits the mRNA and protein expression of VEGF, VEGFR2 and bFGF

**Figure 4:**
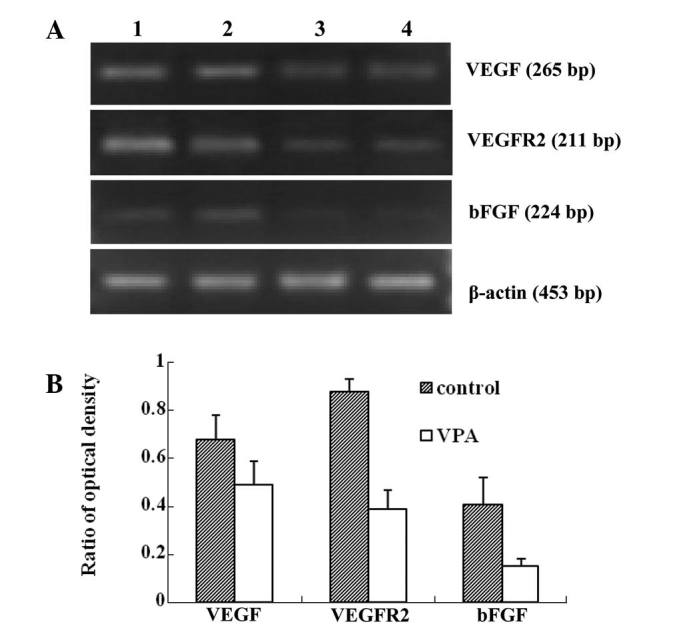
(A and B) Effects of VPA on mRNA expression of VEGF, VEGFR2 and bFGF. mRNA expression was detected by semi-quantitative RT-PCR, and PCR products were separated on agarose gels. Lanes 1 and 2, control groups; lanes 3 and 4, VPA-treated groups; VPA, valproic acid; VEGF, vascular endothelial growth factor; bFGF, basic fibroblast growth factor; VEGFR2, VEGF receptor 2.

**Figure 5:**
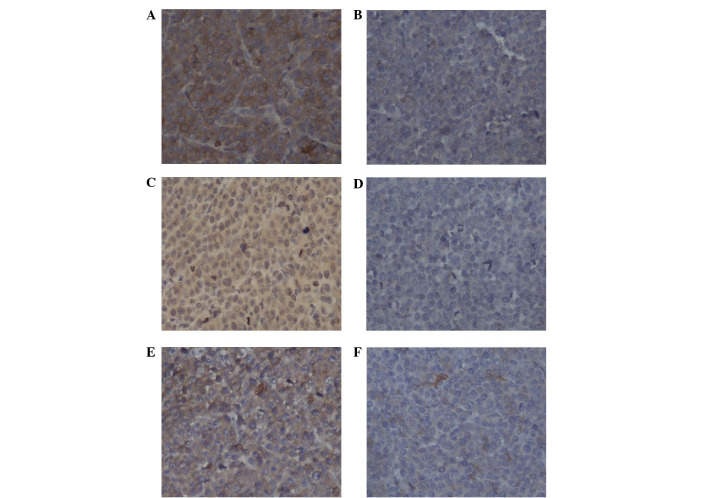
Expression of (A and B) VEGF, (C and D) VEGFR2, and (E and F) bFGF using immunohistochemical analysis (x200). (A, C and E) Control groups. (B, D and F) VPA-treated groups. VEGF, vascular endothelial growth factor; bFGF, basic fibroblast growth factor; VPA, valproic acid; VEGFR-2, VEGF receptor-2.

The mechanisms underlying the VPA-induced inhibition of angiogenesis were studied further in the mice transplanted with Kasumi-1 cells. The mRNA and protein expression levels of VEGF, VEGFR2 and bFGF, which have been reported to be involved in tumor angiogenesis, were examined (9,10). RT-PCR demonstrated that the mRNA levels of *VEGF*, *VEFGR2* and *bFGF* were significantly downregulated in the VPA group compared with those in the control group (Fig. 4). The protein expression of VEGF, VEFGR2 and bFGF was detected using immunohistochemistry (Fig. 5). Consistent with the mRNA levels, the protein expression of VEGF, VEFGR2 and bFGF was suppressed in the VPA group compared with the control group. These results indicated that VPA inhibited tumor angiogenesis most likely through its inhibition of VEGF, VEFGR2 and bFGF.

### VPA inhibits HDAC activity and increases acetylation of histone H3

**Figure 6:**
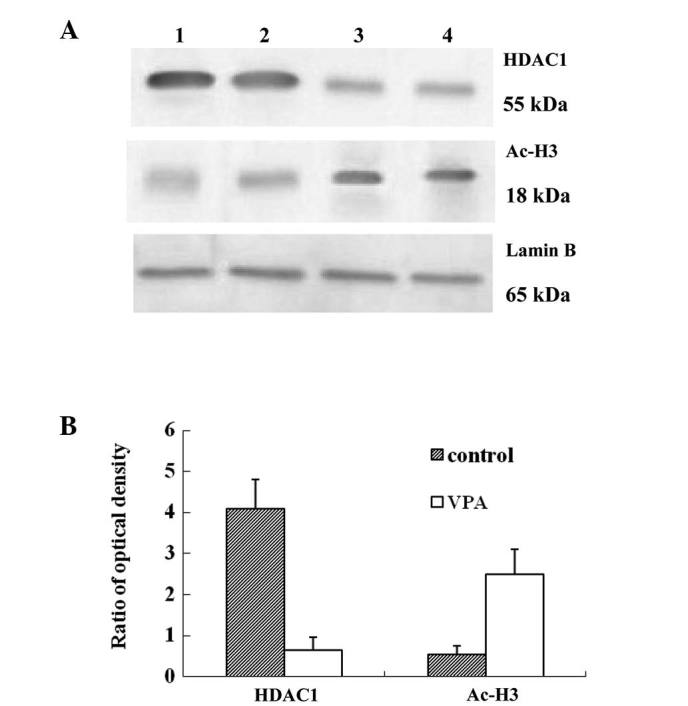
VPA inhibits the protein expression of HDAC1 and increases histone H3 acetylation. Nuclear proteins were extracted from tumors in the control and VPA-treated mice. Nuclear proteins of HDAC1 and acetylated histone H3 (Ac-H3) were immunoblotted using specific antibodies. Lamin B was used as a loading control. Lanes 1 and 2, control groups; lanes 3 and 4, VPA-treated groups; VPA, valproic acid; HDAC, histone deacetylase.

As it has already been shown that the histone acetylation of the VEGF promoters regulates VEGF protein expression (11), the present study investigated the effects of VPA on HDAC activity by detecting the nuclear expression of HDAC1 and the acetylation of histone H3 (Fig. 6). Western blotting revealed that the expression of HDAC1 was downregulated, while histone H3 acetylation was increased in the VPA group compared with the control group, indicating that VPA increased the acetylation of histone H3 via the inhibition of HDAC.

### VPA enhances the accumulation of hyperacetylated histone H3 on VEGF promoters

**Figure 7:**
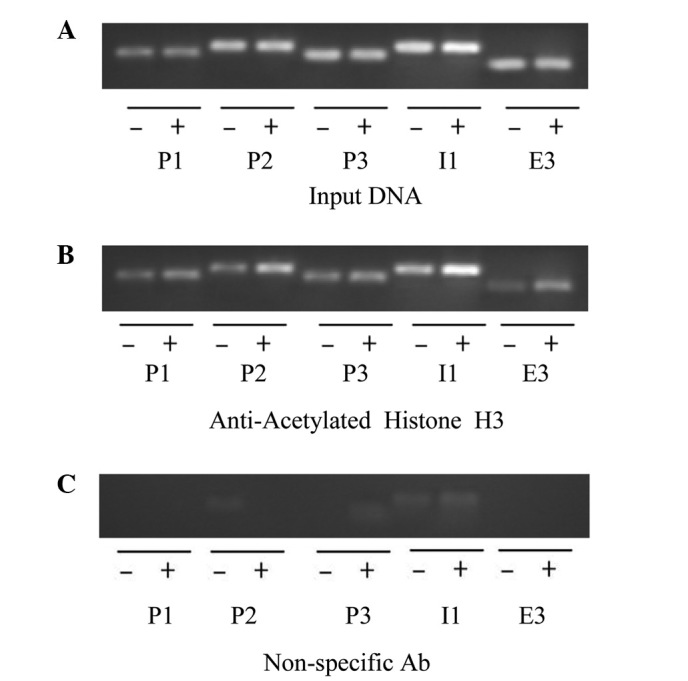
Effect of VPA on the histone H3 acetylation at the promoter of VEGF. Chromatin immunoprecipitation assay was performed using (A) antibodies against RNA polymerase as a positive control, (B) antibodies against acetylated histone H3 and (C) non-specific mouse IgG as a negative control. qPCR was performed with five pairs of primers specific for the *VEGF* promoter (P1, P2, P3, I1 and E3). −, control group; +, VPA-treated group; VPA, valproic acid; VEGF, vascular endothelium growth factor; qPCR, quantitative PCR.

To investigate whether VPA induced the hyperacetylation of histone H3 at the *VEGF* promoter, a ChIP assay was performed using anti-Ac-H3 antibodies to precipitate chromatin from Kasumi-1 cell-induced tumors (Fig. 7). Anti-Ace-H3 antibodies enriched more *VEGF* promoter DNA fragments in the VPA group than in the control group. By contrast, non-specific IgG antibodies did not precipitate *VEGF* promoter DNA in either the VPA group or the control group. Anti-polymerase II antibodies, which served as a positive control, precipitated similar quantities of *VEGF* promoter DNA in the VPA and control groups.

## Discussion

In the present study, a mouse model of leukemia was established using Kasumi-1 cells, which are human acute myeloid leukemia cells with an 8;21 chromosome translocation. Kasumi-1 cells express the AML-1/ETO fusion protein and are ideal for the study of AML with t(8;21) (17). The *in vitro* study by Liu *et al* (18) in Kasumu-1 cells revealed that VPA had pronounced antileukemic effects that were associated with an inhibition of the protein expression of HDAC in the nuclei, a disruption of the physical interaction between AML1/ETO and HDAC1, an increase in their dissociation from the promoters of AML1/ETO target genes and a translocation of these nuclear proteins to the perinuclear region. In agreement with these reported *in vitro* antileukemic effects of VPA, the present *in vivo* study demonstrated that intraperitoneal injections of VPA reduced tumor growth in mice implanted with Kasumi-1 cells, and the tumor weights and volumes were significantly smaller in the VPA group compared with those in the control group.

It has been reported that AML with t(8;21) and Kasumi-1 cell lines demonstrate enhanced expression of VEGF and VEGFR2, which is associated with the growth of Kasumi-1 cells and the angiogenesis of AML (19,20). Blockade of the VEGFR2 signaling pathway inhibits the growth of Kasumi-1 cells (20). Furthermore, several *in vivo* and *in vitro* studies have revealed that HDAC inhibitors, including NaB, SAHA, TSA and FK228, inhibit tumor angiogenesis via downregulation of the mRNA and protein expression of VEGF (21–24). In addition, our previous study revealed that VPA exerted antitumor effects on Kasumi-1 cells, most likely through the downregulation of the mRNA and protein expression of VEGF, VEGFR2 and bFGF (14). In the present study, the effects of VPA were directly tested on tumor angiogenesis in mice transplanted with Kasumi-1 cells by measuring the MVD with CD34 immunostaining. VPA was observed to decrease the MVD and downregulate the VEGF, VEGFR2 and bFGF mRNA and protein levels compared with the control. Thus, the present study provided direct evidence that VPA inhibited tumor angiogenesis in mice transplanted with Kasumi-1 cells.

Several mechanisms have been reported to be involved in HDAC inhibitor-induced anti-angiogenesis, including the inhibition of HDAC activity, the overexpression of HIF-1α, the downregulation of tumor suppressor genes, including VHL and p53, and the downregulation of VEGF expression (25–27). In the present study, VPA, a HDAC inhibitor, was detected to downregulate HDAC protein expression and increase the acetylation of histone H3 in Kasumi-1 cell-induced tumors. In addition, VPA enhanced the accumulation of hyperacetylated histone H3 on the *VEGF* promoters. However, it remains unclear how histone acetylation inhibits gene transcription. There are two possible mechanisms underlying the regulation of gene transcription by histone acetylation: i) Histone has several acetylation sites, and the acetylation state of each lysine is different in each site. It has been reported that the patterns of histone acetylation, which are termed the ‘histone code’ reveal the sign of transcription (28); and ii) histone acetylation by HDAC inhibitors induces alterations in the chromatin structure, which may lead to the disruption of the binding of transcription factors to gene promoters. It has been reported that VPA may alter the chromatin structure by regulating chromatin modulation proteins (29). Although the mechanism underlying the alteration of the chromatin structure by histone acetylation has not been clarified, it may be important to explain how HDAC inhibitors regulate gene expression.

In conclusion, VPA inhibits tumor growth and tumor angiogenesis in mice implanted with Kasumi-1 cells. This antitumor effect of VPA is possibly due to the inhibition of VPA on the expression of angiogenic factors. In addition, VPA may increase the accumulation of acetylated histones on the promoters of the genes, which may contribute to the regulation of the expression of angiogenic factors.

## Figures and Tables

**Table I:** Volume and weight of tumors following sacrifice of the mice.

Group	n	TV, mm^3^	Tumor weight, g	RTV	IR, %
Control	6	2235.0±360.21	1.57±0.25	9.50±2.13	
VPA	6	699.4±271.01^a^	0.46±0.17^a^	4.06±1.05^a^	57.25

a P<0.01 vs. control.

TV, tumor volume; RTV, relative tumor volume; IR, inhibition rate; VPA, valproic acid.

## Abbreviations

HDAC: histone deacetylase
VPA: valproic acid
MVD: microvessel density
AML: acute myeloid leukemia
VEGF: vascular endothelial growth factor
